# Daytime warming has stronger negative effects on soil nematodes than night-time warming

**DOI:** 10.1038/srep44888

**Published:** 2017-03-20

**Authors:** Xiumin Yan, Kehong Wang, Lihong Song, Xuefeng Wang, Donghui Wu

**Affiliations:** 1 School of Geography and Tourism, Guizhou Education University, Guiyang 550018, P. R. China; 2 Key Laboratory of Wetland Ecology and Environment, Northeast Institute of Geography and Agroecology, Chinese Academy of Sciences, Changchun 130012, P. R. China; 3 College of Resources and Environmental Science, Chongqing University, Chongqing 40044, P. R. China; 4 College of Agriculture, Guizhou University, Guiyang 550025, P. R. China; 5 School of Chinese Medicinal Materials, Jilin Agricultural University, Changchun 130117, P. R. China

## Abstract

Warming of the climate system is unequivocal, that is, stronger warming during night-time than during daytime. Here we focus on how soil nematodes respond to the current asymmetric warming. A field infrared heating experiment was performed in the western of the Songnen Plain, Northeast China. Three warming modes, i.e. daytime warming, night-time warming and diurnal warming, were taken to perform the asymmetric warming condition. Our results showed that the daytime and diurnal warming treatment significantly decreased soil nematodes density, and night-time warming treatment marginally affected the density. The response of bacterivorous nematode and fungivorous nematode to experimental warming showed the same trend with the total density. Redundancy analysis revealed an opposite effect of soil moisture and soil temperature, and the most important of soil moisture and temperature in night-time among the measured environment factors, affecting soil nematode community. Our findings suggested that daily minimum temperature and warming induced drying are most important factors affecting soil nematode community under the current global asymmetric warming.

Warming of the global land surface, due to the rapid increasing of greenhouse gases, is an indisputable fact. Temperature increased by 0.74 °C over the last century has been documented, and the temperature further increased at a rate of 1–3 °C by 2100 was predicted^1^. A faster and higher warming during the night than the day, i.e. asymmetric warming, was recognized by long-term meteorological observations and recent climate change scenarios predictions^1^,^2^,^3^. This asymmetric warming is expected to affect the ecological processes of aboveground ecosystem, e.g. plants and their insect herbivores and terrestrial ecosystems carbon budgets^4^,^5^. Here raise the question how asymmetric warming will affect the structure and function of underground ecosystems. However, few studies have to date been carried out to examine the influence of the observed asymmetric warming on belowground ecosystems and much remains unknown.

Terrestrial nematodes are an important component of soil fauna and key agents of underground ecosystem’s ecological processes, especially the decomposition of soil organic matter^6^,^7^. Furthermore, nematodes are sensitive to environmental disturbances and are suitable indicators of environmental stress^6^,^8^,^9^. It’s very likely that any alterations of nematode communities induced by warming may have a considerable influence on soil ecosystem function, especially though the changes of soil food webs^10^. Knowledge of the warming controls on soil nematode community is thus needed for improving our understanding soil ecosystem responses to climate change and how to manage the ecosystem sustainability.

Temperature has been evidenced as an important environmental factor affecting nematode community^11^,^12^,^13^,^14^. However, contradictory responses of soil nematodes to temperature changes in field studies were published. It was proposed that with soil temperature increased by 1 °C could have only small effects on soil nematodes^13^. The same marginally effect of warming on soil nematode density were reported in recent publications too^10^,^15^,^16^. In addition, negative effect^11^ or positive effect^14^,^17^ of warming on nematode density were reported also. Soil nematodes could be impacted not only directly by warming, but also indirectly via changed soil micro-environment (e.g. drying) or plant functional groups caused by warming^15^,^18^. Bakonyi *et al*.^10^ ascribed these contradictory results to the different experimental circumstances, nematode species composition, soil type, soil moisture, and so on. Although the contradictory results of different publications, all of them indicated the species specific of nematode genera to warming. Most of these experiments have been conducted under symmetric (diurnal constant) warming. However, responses of soil nematodes to the observed asymmetric warming have been inadequately studied.

In the present study, we examine the effect of asymmetric warming (i.e. daytime warming, night-time warming and diurnal warming) on soil nematodes in field, using infrared heaters to simulate climate warming. We hypothesized that soil nematode would response differently to asymmetric warming and drying caused by warming could be an important factor affecting soil nematode community as the semiarid condition of the study site.

## Results

### Soil temperature and moisture

Warming significantly increased soil temperature (*p* < 0.05) and decreased soil moisture (*p* < 0.05) (Table 1, Supplementary Figs 1 and 2). The maximum temperature increase and the maximum moisture decrease were observed in diurnal warming treatment (24 hW), with an average increase of 1.3 °C, 1.9 °C and 1.5 °C for average daytime temperature (T_day_), average night-time temperature (T_night_) and average diurnal temperature (T_diurnal_), respectively, and a decrease of 14.8% of soil moisture (Table 1). Daytime warming (DW) and night-time warming (NW) significantly increased 0.6 °C and 0.6 °C of the T_day_ and T_night_, respectively. DW and NW significantly decreased soil moisture by 9.0% and 2.2%.

### Soil nematodes

Nematodes were dominated by bacterial feeders and fungal feeders, accounting for 89.4% of the total. *Acrobeloides* was the most abundant nematode, which accounted for 32–47% of the animal in all plots, regardless of treatment, and *Paraphelenchus* (15–26%) and *Acrobeles* (11–21%) followed in order.

**Figure 1:**
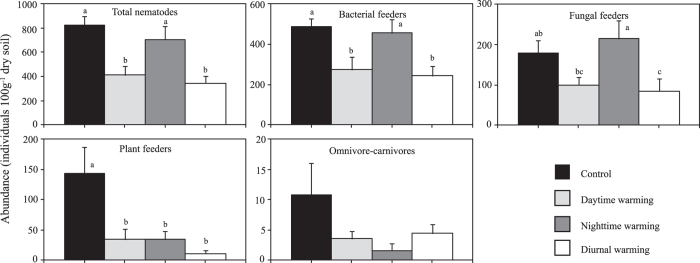
Abundance of the total nematodes (ANOVA, df = 3, F = 9.3, p = 0.00), bacterial feeders (F = 6.1, p = 0.01), fungal feeders (F = 3.9, p = 0.02), plant feeders (F = 6.0, p = 0.01) and omnivore-carnivores (F = 1.5, p = 0.26) in control (C), daytime warming (DW), night-time warming (NW) and diurnal warming (24 hW). Bars with different letter (**a**–**c**) are significant difference based on LSD. Error bars represent standard error (SE).

Nematode populations were significantly affected by asymmetric warming (*p* < 0.05, Fig. 1 and Supplementary Table 2). Total abundance was significantly decreased in DW and 24 hW, while not significant in NW (Fig. 1). Significant differences were found of nematode functional groups and species to asymmetric warming (Fig. 1 and Supplementary Table 2). Bacterivores and fungivores population were significantly decreased in 24 hW, whereas they were not influenced in NW. DW significantly decreased the density of bacterial feeders, whereas not significant for fungal feeders. All of the three warming treatments significantly decreased the density of plant feeders. Because of low abundance and high variation of omnivore-carnivores, effect of warming on this group was not significant (Fig. 1).

**Figure 2:**
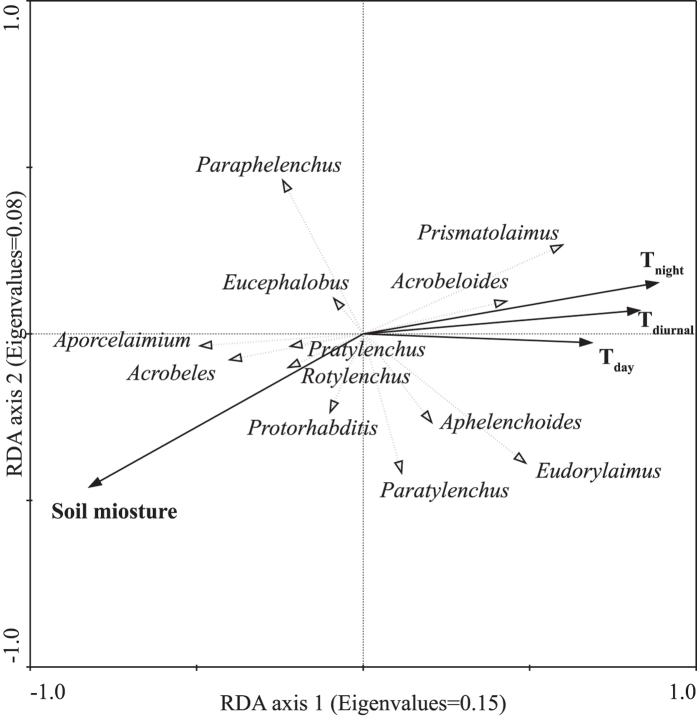
RDA bi-plot of nematode genus and environmental variables. T_night_ is average temperature in night-time. T_day_ is average temperature in daytime. T_diurnal_ is daily (24 hour) average temperature. The species-environment correlations were 0.76 for axis 1 and 0.74 for axis 2. The environmental variables explained 27.9% of the total variance in the species data (Soil moisture: 14.6%, *p* = 0.002; T_night_: 14.4%, *p* = 0.002; T_diurnal_: 13.3%, *p* = 0.004; T_day_: 11.2%, *p* = 0.010).

Redundancy analysis (RDA) diagram indicated an opposite effect of soil temperature and soil moisture on soil nematode community, and the measured soil temperature and moisture together explained 16.9% of the variance in nematode community (*p* < 0.05, Fig. 2). Soil moisture was the most important among the measured environmental variables, which alone explained 8.1% of the variation. In addition, T_night_ also explained a significant portion of the variation in nematode community (7.8%, *p* < 0.05, Fig. 2).

## Discussion

We simulated climate asymmetric warming by experimentally heating in day and/or night. Warming treatments changed soil microclimate (soil temperature and moisture). In agreement with our hypothesis, results showed different effects of daytime warming and night-time warming on soil nematodes. We highlight that warming induced drying and daily minimum temperature are most important factors affecting soil nematodes under the current asymmetric warming in Songnen grassland.

As far as we known, there were only two studies studied the effect of night-time warming on soil nematodes, in which showed the same results with the present study. These authors suggested that, the ebb and flow of the different nematode genera maintained the stable population size under NW^10^,^16^. It was evidenced that compared to control, the abundance of *Acrobeles, Aphelenchoides* and *Rotylenchus* decreased, whereas the abundance of *Acrobeloides* and *Paraphelenchus* increased under NW (Supplementary Table 1). Comparable to the results by Simmons *et al*.^11^ who reported that a long-term diurnal warming by OTC in Antarctica reduced soil nematode populations, and these authors suggested both the direct and indirect effects by warming induced altering species-specific habitat suitability for soil biota. While, more publications reported positive effects of warming on nematode density^14^,^17^. Warming could induce a greater reproduction rate and result in a higher population density^14^. Additionally, soil warming enhanced the microbial biomass^19^, which may in turn benefit nematode population, especially microbial-feeding nematodes. All the warming treatments significantly increased soil temperature, regardless of T_day_, T_night_ or T_diurnal_ (Table 1). However, soil warming did not affect (NW), even decreased (DW and 24 hW), soil nematode populations in the present study (Fig. 1). This indicated that temperature is not the determination factor for soil nematode community in the study area.

It is well known that soil temperature and moisture content are among the main abiotic factors which directly or indirectly influence the activity, distribution and population dynamic of nematodes in the soil^20^,^21^,^22^. The increase of soil temperature coincided with a significant decrease of soil moisture (Table 1). Free-living nematodes were highly dependent on free water in soil for locomotion and biological activity, decreasing soil moisture content was expected to be a detrimental factor^18^,^23^,^24^. It was reported that, warming induced water shortage in soil reduced the density of nematodes in a multi-factor global change experiment in grassland^18^.

According to the differences in nematode responses between DW and NW, the temperature increase rates were almost same, but the soil moisture was greatly different, suggesting that warming-induced soil moisture change was the major driving factor of soil nematodes responses, not the warming. As a result, the sharp decrease of soil moisture caused by warming could be the reason of the decreasing of soil nematodes in DW and 24 hW in the present study.

In field experiments, it is seldom possible to explain and separate the responses of soil nematodes to warming and drying, because of the different responses of nematode species to changes in temperature and moisture^10^,^22^. Ordination analysis could be one solution of the issue. In the present study, results of RDA indicated the opposite effect of soil temperature and soil moisture on soil nematode community (Fig. 2). As discussed above, soil temperature positively affect soil nematodes, however, warming induced drying negatively affect soil nematodes. The results of asymmetric warming on soil nematodes possibly was the balance of these two conversely issue.

Our data showed that warming decreased the abundance of plant feeders due to significantly lower abundance of *Rotylenchus*, indicating the warming sensitivity of this fauna. It’s common that most nematode species were sensitive to environmental changes and the narrow temperature/moisture range often determine the distribution of a particular species^25^,^26^. The decrease of plant feeder possibly could increase the ratio between microbial feeding and plant feeding nematodes density [(FF + BF)/PF], as showed in previous publications^15^,^16^,^27^. The warming induced disproportionate responses of nematodes functional groups could have a substantial impact on soil food web dynamics and ecosystem functioning^28^,^29^. The significant declining of plant feeders by warming in this study also indicated that warming induced soil drying was fatal for plant feeding nematodes in semiarid-arid system.

## Conclusions

The present study was a complementary research in global change biology/ecology, especially in the ecological influence of asymmetric warming. The changes of soil nematodes were due to the integrated effects of temperature and soil moisture changes caused by warming. Warming in the daytime had a stronger negative effect on soil nematodes than warming in the night-time, which was the main finding of the present study. As eigenvalues of RDA axes were not remarkably high, soil nematodes could be affected by other environmental variables, which were not included in our present study. Nevertheless, RDA indicated the significant importance of soil moisture as well as soil temperatures in the night-time on soil nematode community. This suggested, under the current global asymmetric warming and regional characteristics of global warming^1^, the importance of daily minimum temperature and warming induced drying in underground ecosystem.

## Methods

### Study site

The research site was located in the Ecological Research Station of Grassland Farming, Changling, Chinese Academy of Sciences, northeast China (44°33′N, 123°31′E, 145 m a.s.l.). The climate is temperate semi-arid continental monsoon. The annual average temperature is about 4.9 °C (with an average of 23 °C in July and −15 °C in January). The frost-free period lasts 140 days per year. The annual precipitation is 300–450 mm, of which 70–80% occurred between July and September, with the annual evaporation of 1500–2000 mm.

### Experimental design

The field experiment included three warming treatments: daytime warming (DW, 06:00–18:00), night-time warming (NW, 18:00–06:00) and diurnal warming (24 hW), and one ambient control (C). A randomized block design with 6 replicates was used. A total of 24 plots (3 m × 4 m in size for each) were arranged in a 4 × 6 matrix. The distance between the adjacent plots was 3 m. Plots were warmed by MSR-2420 infrared radiators (2 KW, Kalglo Electronics, Bethlehem, Pennsylvania, USA), which was suspended 2.25 m above the ground. For control plots, ‘dummy’ heaters with the same shape, size and installation as the heaters in the warming plots were set. Warming started on 1 June 2012 and ended on 31 August 2012. *Astragalus adsurgens* Pall. was planted in the experimental plots. Sensors probes of temperature and humility were buried 5 cm below the soil surface in the centre of 3 random selected plots for each treatment. Soil temperature and humidity were auto-recorded per hour.

### Soil sampling and nematode analysis

Nematodes samples were sampled on 31August, 2012. A mixed soil sample of three soil cores (diameter: 5 cm, depth: 5 cm) was collected per plot. 100 g fresh subsamples were used to extract nematodes. A modified sucrose centrifugal flotation method was used for nematode extraction immediately after sampling^30^. The extracted nematodes were preserved in 4% formaldehyde. One tenth and an average of 150 nematode individuals of each sample was identified and counted under microscope Nikon 80i. Nematodes were identified to the genus level according to the keys/procedures of Bongers^31^. The functional groups of nematodes (i.e. bacterial feeders, fungal feeders, plant feeders and omnivore-carnivores) were divided according to Yeates *et al*.^32^. Nematode density was expressed as individual numbers 100 g^−1^ dry soil.

### Statistical analysis

Soil average daytime temperature (T_day_, 06:00–18:00), soil average night-time temperature (T_night_, 18:00–06:00) and soil average diurnal temperature (T_diurnal_) and soil moisture were calculated separately. Densities of total nematodes and four functional groups were calculated (individuals 100 g^−1^ dry soil).

Repeated measure ANOVA was used to test the effect of asymmetric warming treatments on soil temperature (T_day_, T_night_ and T_diurnal_) and soil moisture. ANOVA was applied to test the effect of warming on nematodes (total abundance and density of four functional groups). Post-hoc comparisons using Turkey’s LSD test were followed. The data for nematode density were log (n + 1) transformed before analysis. Redundancy analysis (RDA) and an ordination bi-plot were used to visualize the correlation between nematodes and soil temperature and moisture. The significance of the environmental parameters was analyzed by Monte Carlo permutation tests (499 permutations). All analysis were performed with R3.1.2 using the *vegan* package^33^, at a significant level α = 0.05.

## Additional Information

**How to cite this article**: Yan, X. *et al*. Daytime warming has stronger negative effects on soil nematodes than night-time warming. *Sci. Rep.* **7**, 44888; doi: 10.1038/srep44888 (2017).

**Publisher's note:** Springer Nature remains neutral with regard to jurisdictional claims in published maps and institutional affiliations.

## Supplementary Material

Supplementary Materials

## Figures and Tables

**Table 1:** Statistic results of asymmetric warming on soil temperature (Mean ± SD) and soil moisture (Mean ± SD).

Treatment	Soil temperature (°C)			Soil moisture (%)
	Day time^1^	Night time^2^	Diurnal^3^	
Control	23.1 ± 2.5 d	22.0 ± 2.5 c	22.6 ± 2.4 c	30.8 ± 10.2 a
Daytime Warming	23.7 ± 2.5 b	22.3 ± 2.5 bc	23.0 ± 2.5 b	21.8 ± 6.9 c
Nighttime Warming	23.4 ± 2.4 c	22.6 ± 2.4 b	23.0 ± 2.3 b	28.6 ± 15.4 b
Diurnal Warming	24.4 ± 2.3 a	23.9 ± 2.5 a	24.1 ± 2.3 a	16.0 ± 8.0 d
F^4^	44	64	175	454
*P*^4^	<0.01	<0.01	<0.01	<0.01

Values in each column followed by different letter (a–d) are significant difference based on LSD.

^1^Day time means 06:00–18:00.

^2^Night time means 18:00–06:00.

^3^Diurnal means 06:00–06:00.

^4^Repeated measures ANOVA.
