# Erratum: Daytime warming has stronger negative effects on soil nematodes than night-time warming

**DOI:** 10.1038/srep46881

**Published:** 2017-08-29

**Authors:** Xiumin Yan, Kehong Wang, Lihong Song, Xuefeng Wang, Donghui Wu

Scientific Reports 7: Article number: 44888; 10.1038/srep44888 published online: 03 20 2017; updated: 08 29 2017.

This article was published twice in error during a change in production systems. The publisher apologizes to the authors and readers for the error. When citing this work, please refer to the original version.^1^

## References

[b1] Yan X. . Daytime warming has stronger negative effects on soil nematodes than night-time warming. Sci. Rep. 7, 108 (2017). 10.1038/s41598-017-00218-4. 28273897 PMC5428061

